# Predicting ground reaction forces of human gait using a simple bipedal spring-mass model

**DOI:** 10.1098/rsos.211582

**Published:** 2022-07-27

**Authors:** Michael Mauersberger, Falk Hähnel, Klaus Wolf, Johannes F. C. Markmiller, Alexander Knorr, Dominik Krumm, Stephan Odenwald

**Affiliations:** ^1^ Chair of Aircraft Engineering, Technische Universität Dresden, 01062 Dresden, Germany; ^2^ Elbe Flugzeugwerke GmbH, 01101 Dresden, Germany; ^3^ Department of Sports Equipment and Technology, Chemnitz University of Technology, 09107 Chemnitz, Germany

**Keywords:** human gait, bipedal spring-mass model, structural design, ground reaction force prediction

## Abstract

Aircraft design must be lightweight and cost-efficient on the condition of aircraft certification. In addition to standard load cases, human-induced loads can occur in the aircraft interior. These are crucial for optimal design but difficult to estimate. In this study, a simple bipedal spring-mass model with roller feet predicted human-induced loads caused by human gait for use within an end-to-end design process. The prediction needed no further experimental data. Gait movement and ground reaction force (GRF) were simulated by means of two parameter constraints with easily estimable input variables (gait speed, body mass, body height). To calibrate and validate the prediction model, experiments were conducted in which 12 test persons walked in an aircraft mock-up under different conditions. Additional statistical regression models helped to compensate for bipedal model limitations. Direct regression models predicted single GRF parameters as a reference without a bipedal model. The parameter constraint with equal gait speed in experiment and simulation yielded good estimates of force maxima (error 5.3%), while equal initial GRF gave a more reliable prediction. Both parameter constraints predicted contact time very well (error 0.9%). Predictions with the bipedal model including full GRF curves were overall as reliable as the reference.

## 1. Introduction

### 1.1. Designing aircraft structures under user-induced loads

Especially in the aerospace industry, low weight of structural components is of high priority to reduce operating costs. Structural loads need to be specified in detail to design aircraft structures adequately according to certification specifications [1]. Although most exterior parts of aircrafts are designed using common load spectra, there are only few standard load requirements for the interior. However, aircraft users such as passengers and crew members interact almost freely with floorings, panels and other cabin parts and that can generate additional structural loads which are difficult to estimate.

Human gait is an example of common source of user–structure interactions in the aircraft interior. Especially floor panels are subject to high and frequent loading while fulfilling a critical role in the shear stability of the aircraft fuselage. One way to estimate these loads are experimental studies. Since aircraft design wants to find optimal structures for general load cases, many experiments would be necessary. A more elegant way of generating human-induced loads is parametrized human models. These inherently represent consistent movement dynamics while only requiring few input parameters. These models simulate outputs of ground reaction forces (GRF) as well as fundamental gait kinematics which can be incorporated in different load cases, e.g. in static or life cycle design. Experiments are only required here for parameter calibration. Moreover, simulation results from human models can later be included in detailed dynamic structural finite-element simulations, e.g. regarding the loading phase during foot rollover [2]. Together with human walking models, aircraft interior structures may be designed in a general, fast and flexible end-to-end process, which only depends on easily determinable aircraft user-related input parameters.

### 1.2. Using simple bipedal models for ground reaction force prediction

Modelling human gait has already been an object of intensive research over the past decades. Early attempts based on mechanical spring-mass systems were made as early as the 1980s and early 1990s [3–7]. Later, running and walking were generalized using spring-loaded inverted pendulum (SLIP) models [8–10]. All approaches have in common that they use leg springs as a major determinant of shaping GRF [8]. Full & Koditschek [11] introduced the modelling principle of templates and anchors. According to the authors, templates are the simplest models of human locomotion that simplify redundant biomechanical joint and muscle functions. Using the SLIP model as a template, more complex variants assume the role of specific anchors. These take on the task of presenting special features such as roller feet [12–14], swing leg dynamics [15–17], additional leg joints [18] or trunk segments [15].

Various few studies dealt with the prediction of GRF by means of a simple bipedal model. Buczek *et al*. [19] applied a SLIP model directly to human gait to estimate characteristic parameters of gait speed and GRF. The authors used a model with telescoping legs as a crucial feature to reproduce the well-known double-hump curve of vertical GRF. Lipfert *et al*. [20] used the simplest SLIP template of Geyer *et al*. [9] in order to predict GRF and kinematics in comparison with experimental data for walking and running. They found good qualitative predictions of the SLIP model but considerably underestimated contact times. Especially for walking, only few experiments could be modelled due to incompatible parameter values. Ryu & Park [21] investigated the quality of GRF prediction using different amounts of experimental data that were available. The model in use had linear leg springs and described the temporal movement of the centre of pressure (CoP) based on the results of Jung & Park [22]. Wearable motion monitoring devices were used to measure important gait parameters, such as gait speed and the number and frequency of steps. Model locomotion and ground contact were optimized to match GRF. Furthermore, non-conservative and active bipedal models were investigated for GRF prediction [13,23,24]. All these models need additional energy input to move periodically and differ significantly from passive SLIP models.

### 1.3. Objective and concept of the present paper

The main objective of this paper was to establish a method for predicting user-induced load cases in the overall end-to-end process for the design of aircraft interior structures. Therefore, a passive walking SLIP model was used as a simple GRF predictor. In this design process only easily estimable user-related parameters are supposed to be necessary as inputs. The passive SLIP model of Whittington & Thelen [12] with circular roller feet was applied here in order to consider the aspect of relative CoP motion during foot rollover. At the same time, no unknown model input parameters had to be added. The advantage of this model is that changes of the GRF location on the structure can be generated. It is believed that these changes have a strong influence on the structural stresses due to the small dimensions of the floor panels.

As it is known from previous experimental studies, modelling real walking dynamics by SLIP models is limited [20]. However, current literature lacks both a clear quantification of these limitations and a method to facilitate a reliable SLIP model-based GRF prediction process. These should compensate for limitations and should not need further experimental data. That leads to the following research questions: is the GRF prediction of the SLIP template proposed by Whittington & Thelen [12] limited in the same way as that of the simplest SLIP template [9,20]? How can SLIP model limitations be quantified and incorporated into compensatory means for a reliable GRF prediction without the need for further experimental data?

**Figure 1.**
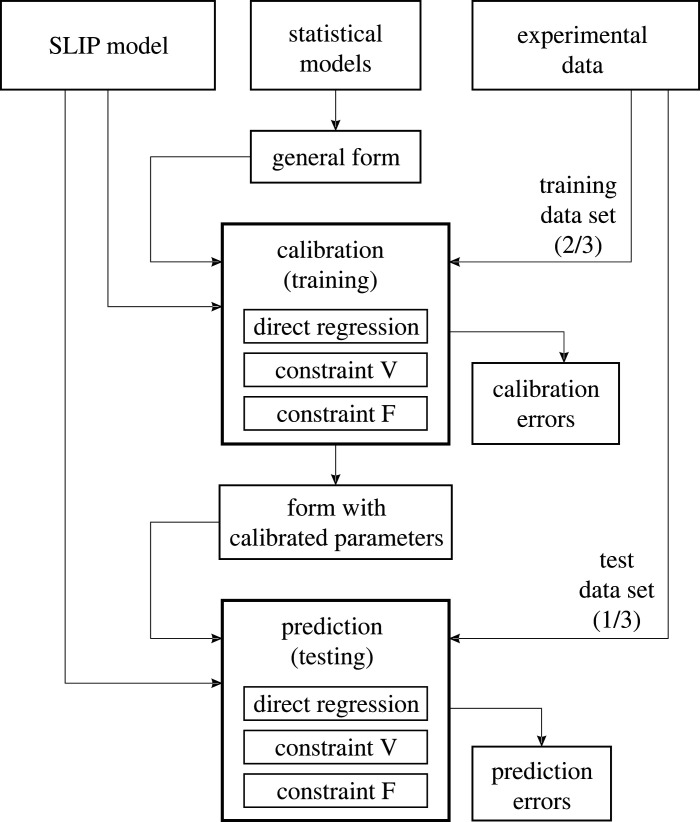
Flowchart for the calibration and prediction steps used in this paper. The amount of experimental data is divided in two thirds for training (calibration) and one third for testing (prediction). Model and experimental results are compared via the bipedal model considering two parameter constraints V and F. Direct regression models serve as a reference for prediction. Statistical models are calibrated by experimental training data via regression analysis. Prediction errors are evaluated by experimental test data.

To address these aims, this study was conceived in two main steps: (i) model development with calibration and (ii) GRF prediction as the application in the design process. Figure 1 shows the study's concept in a flowchart. Experiments for comparison were conducted within an aircraft interior mock-up representing common environmental conditions of the gait interactions. Two parameter constraints for the comparison between experimental data and the SLIP model simulation were proposed. These are thought to be necessary for a representative fit between SLIP model and experiment, which is kept explicitly explainable due to minimal usage of parameter optimization. In the first step, the prediction model was developed and limitations were identified. Calibration of additional regression models should compensate for these limitations and facilitate the GRF prediction without further experimental data. The SLIP model together with the compensatory regression models is called assisted SLIP (SLIP+) model. In the second step, GRF predictions were carried out by means of the SLIP+ model. Sample GRF were generated for different aircraft user classes to demonstrate the functionality of the overall load case prediction process. In order to assess the capabilities of the proposed method, direct statistical regression models were used as a reference for GRF prediction without SLIP model simulation. It is believed that SLIP+ prediction should raise similar or lower errors than the reference in order to be promisingly applied to the structural design process.

This paper offers a thorough investigation regarding limitations of the passive SLIP model proposed by Whittington & Thelen [12], which has not been carried out before. Parameter constraints as an explicit concept is newly introduced in order to focus on the SLIP model locomotion which effectively represents experimental data. Furthermore, additional statistical models enable the SLIP+ model to predict accurate and fully dimensional GRF data. SLIP models can produce full GRF curves while estimating basic walking kinematics consistently, e.g. leg orientation and CoP motion. Hence it is believed that they are generally superior to direct statistical regression of single parameters as it is done in the reference prediction. Table 1 contains all symbols introduced in this paper.

**Table 1.** Symbols introduced within this paper.

symbol	description	unit
$A_{tc}$	contact time factor	none
$A_{lh}$	leg-to-body ratio	none
$b_{\mathrm{act}}$	switch for leg activation	none
$F_{v,\max}$	maximum vertical GRF	N
$F_{v,\min}$	trough value in vertical GRF	N
$F_{ap,\max}$	maximum absolute anteroposterior GRF (walking direction)	N
$f_{s}$	step frequency	s^−1^
$f_{c}$	contact frequency	s^−1^
$h_{0}$	body height	m
$k_{0}$	model leg stiffness	N m^−1^
$l_{s}$	step length	m
$l_{0}$	model leg length	m
$m_{0}$	body mass	kg
$p_{\varphi c}$	model step parameter	none
$R^{2}$	coefficient of determination (linear relationship)	none
$r_{0}$	model roller foot radius	m
s	model leg compression	m
$t_{c}$	contact time	s
$t_{s}$	step duration	s
$v_{G}$	gait speed	m s^−1^
$x_{0}={\left(x_{0},y_{0}\right)}^{T}$	model CoM coordinates	m
$z_{p}$	model state at Poincaré section	m, m s^−1^
$\varphi_{TD}$	model touchdown angle	degrees (°)
$\tilde{\left(\cdot \right)}$	normalized value	none
${\left(\cdot \right)}^{0}$	value at vertical leg condition	diverse

## 2. Methods

### 2.1. Modelling human gait

**Figure 2.**
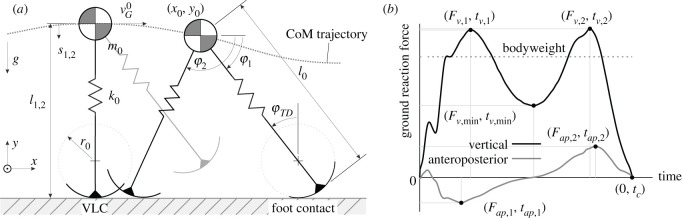
(*a*) Parameter definition of bipedal spring-mass model with roller feet, shown while moving from vertical leg condition to foot contact. (*b*) Definition of vertical and anteroposterior ground reaction force parameters.

The SLIP model of Whittington & Thelen [12] with added roller feet is used for GRF prediction throughout this paper. It is modelled by a spring-mass system consisting of a mass point at the human centre of mass (CoM) and two massless linear spring legs with total rest length $l_{0}$ and constant stiffness $k_{0}$. The SLIP template from Geyer *et al*. [9] was extended to include circular roller feet with constant radius $r_{0}$ (cf. figure 2*a*). The advantage of this variant of CoP excursion is that a confirmed value of $r_{0}/l_{0}\approx 0.3$ exists in literature [25,26] and no additional unknown model parameter has to be introduced.

The SLIP model was reconfigured to global Cartesian CoM coordinates to facilitate the generalization of single and double support phases to one differential equation with activation parameters [9]. With it, a general and simple formulation of the equations of motion is achieved. Foot contact (FC) and foot-off (FO) conditions indicate whether the model simulation switches from single to double or from double to single support, respectively. The parameter vector $b_{\mathrm{act}}$ indicates this phase change by taking the values 0 (deactivation) or 1 (activation) for each of the legs. The equations of motion follow directly from Lagrange's equations of the second kind:

2.1 $$0={\ddot{x}}_{0}+\frac{k_{0}}{m_{0}}\mathrm{diag}\left(b_{\mathrm{act}}\right)\left(J_{x_{0}}\left(s\right)s\right)+g,$$

where

2.2 $$\begin{array}{rl} x_{0} & ={\left(x_{0},\;y_{0}\right)}^{T},\\ b_{act} & ={\left(b_{act,1},\;b_{act,2}\right)}^{T},\\ s & ={\left(s_{1},\;s_{2}\right)}^{T},\\ g & ={\left(0,\;-g\right)}^{T},\\ J_{x_{0}}\left(s\right) & ={\left(\frac{\partial }{\partial x_{0}},\;\frac{\partial }{\partial y_{0}}\right)}^{T}s^{T}. \end{array}\}$$

The vector $x_{0}$ describes the position of the CoM, ***s*** defines the spring compression of the individual legs, and the vector ***g*** represents the acceleration due to gravity (cf. figure 2*a*). To estimate the influences of the leg springs on the CoM, $J_{x_{0}}$ specifies the Jacobian matrix with respect to the CoM coordinates $x_{0}$. Indices 1 and 2 indicate the leg numbers.

The model parameters were normalized as proposed by Hof [27] (cf. table 2). In this way, the simulation results are generally valid and can be adapted to the properties of each aircraft user-related load case by re-dimensioning. The tilde $\tilde{\left(\cdot \right)}$ indicates normalized variables.

**Table 2.** Reference values for the normalization of model dimensions according to Hof [27].

model dimension	reference value	concerned model parameters
length	$l_{0}$	$x_{0}$, $l_{0}$, s, $r_{0}$
mass	$m_{0}$	$m_{0}$
acceleration	g	${\ddot{x}}_{0}$, g
time	$\sqrt{l_{0}/g}$	$t_{s}$, $t_{c}$, $f_{s}$, $f_{c}$
velocity, speed	$\sqrt{l_{0}\;g}$	${\dot{x}}_{0}$, $v_{G}$
force	$m_{0}\;g$	$F_{v,\max}$, $F_{v,\min}$, $F_{ap,\max}$
spring stiffness	$m_{0}\;g/l_{0}$	$k_{0}$

Equation (2.1) was solved numerically using the fourth-order Runge–Kutta method. The leg angles $\varphi ={\left(\varphi_{1},\;\varphi_{2}\right)}^{T}$ were obtained from the foot position and the CoM coordinates by a simple iterative Newton–Raphson method. All programming code was implemented in Matlab version 2019b (MathWorks, Natick, MA, USA).

The locomotion of the model starts from the vertical leg condition (VLC) as the initial condition, denoted by ${\left(\cdot \right)}^{0}$, at the single support phase [28] with the initial horizontal speed $v_{G}^{0}$. At each point in time, the algorithm checks whether the FC condition

2.3 $$y_{0}\leq r_{0}+(1-r_{0})\cos\varphi_{TD}$$

is fulfilled (cf. figure 2*a*). The touchdown angle $\varphi_{TD}$ is kept constant for each FC, which results in the start of a new double support with the second leg as the leading leg. Later, the next single support phase begins with the take-off of the trailing leg at the FO condition

2.4 $$y_{0}>r_{0}+(1-r_{0})\sin\varphi_{tr},$$

where $\varphi_{tr}$ denotes the trailing leg angle. No swing leg function was implemented because no leg mass is included. A short delay prevents immediate reactivation of the leg to allow for a non-zero swing phase. The model locomotion stops and becomes invalid if there is a negative CoM height $y_{0}$ or gait speed ${\dot{x}}_{0}$.

Periodic gait patterns were achieved via limit cycle analysis. The VLC serves as the Poincaré section $z_{p}$. Assuming that no energy loss occurs during a step, there are only two independent descriptors for the dynamic system state $z_{p}$. In the present analysis, these are the CoM height and gait speed in the form of $z_{p}={\left(y_{0},\;{\dot{x}}_{0}\right)}^{T}$. The return state $z_{p}^{R}$ is reached after one full step at the new VLC. Periodicity is achieved if the deviation

2.5 $$\Delta z_{p}=\left|z_{p}^{R}-z_{p}^{0}\right|$$

from the initial condition reaches zero. This indicates a fixed point $z_{p}^{*}$ and thus a limit cycle. This condition cannot be exactly fulfilled by numerical calculation. Therefore, $\Delta z_{p}$ should be at least smaller than a small value $\Delta z_{p,\lim}$. Asymptotically stable system behaviour was proven for all fixed points $z_{p}^{*}$ by analysing the dynamic Jacobian matrix $J_{z_{p}^{*}}$. Then, if all eigenvalues are less than one, system motions near $z_{p}^{*}$ are locally stable [28,29]. Appendix A.1 explains the exact calculation method of $J_{z_{p}^{*}}$ as a reference.

The initial condition (VLC) requires the CoM height $y_{0}^{0}$ and the horizontal CoM speed ${\dot{x}}_{0}^{0}=v_{G}^{0}$ to fully describe the state $z_{p}^{0}$. Due to the difficulty of estimating the real $y_{0}^{0}$, this value is expressed as the more accessible GRF trough value $F_{v,\min}$ (cf. figure 2*b*). Previous studies confirm that $F_{v,\min}$ appears to be more concrete than $y_{0}^{0}$ for characterizing gait [30–32]. If $F_{v,\min}$ occurs at the VLC, the normalized force ${\tilde{F}}_{v,\min}$ takes the place of $y_{0}^{0}$ at VLC via

2.6 $${\tilde{F}}_{v,\min}^{0}=\frac{{\tilde{s}}_{1}^{0}}{{\tilde{k}}_{0}}=\frac{1-{\tilde{y}}_{0}^{0}}{{\tilde{k}}_{0}}.$$

The model parameters $\varphi_{TD}$ and $k_{0}$ additionally prescribe the system behaviour during locomotion. Seyfarth *et al*. [33] specified a relationship between these two parameters for running. Here it can be used for walking to scale possible values of $k_{0}$ to a suitable range due to asymptotic behaviour for small $\varphi_{TD}$. The step parameter $p_{\varphi k}$ is introduced as

2.7 $$p_{\varphi k}={\tilde{k}}_{0}(1-{\tilde{r}}_{0})\sin^{2}\varphi_{TD}\approx \mathrm{const}$$

to scale ${\tilde{k}}_{0}\;$to a form that is convenient for the SLIP model with the roller foot radius ${\tilde{r}}_{0}$. A derivation of the proposed calculation method can be found in the appendix A.2.

Vertical and anteroposterior (i.e. walking direction) GRF were simply calculated from the leg spring forces and the respective leg angles via

2.8 $$F_{i}=\left(\begin{array}{c} F_{v,i}\\ F_{ap,i} \end{array}\right)=k_{0}s_{i}\left(\begin{array}{c} \sin\varphi_{i}\\ \cos\varphi_{i} \end{array}\right)\;\;\;\mathrm{with}\;i=1,2.$$

Existing moments in the centre of the foot circle are not eliminated by force translation as suggested by Whittington & Thelen [12].

### 2.2. Measuring ground reaction forces

**Figure 3.**
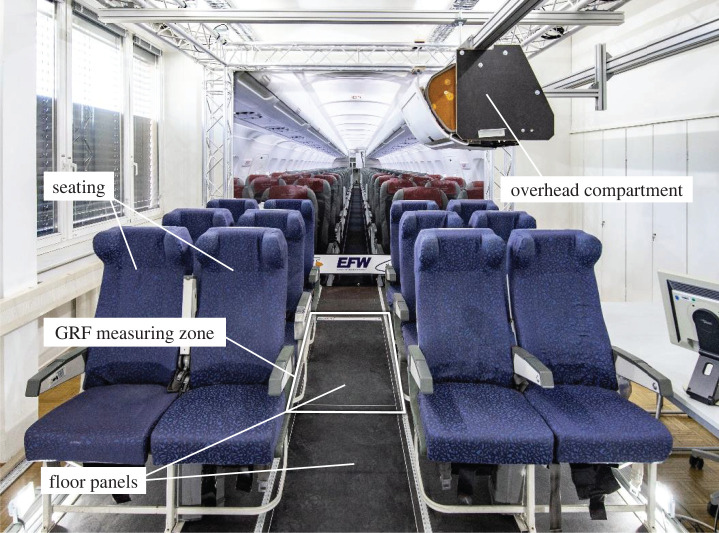
Aircraft interior mock-up representing the corridor in an Airbus A310. The walking direction is parallel to the aisle.

GRF were determined by a gait measurement study for aircraft users walking along the aisle of an aircraft. It was conducted in a mock-up representing the interior of an Airbus A310 aircraft corridor under laboratory conditions (cf. figure 3). Various boundary conditions, i.e. floor panel stiffness, bearing condition, footwear and gait speed, were chosen to emulate the overall influence of the aircraft-related environment on the user–structure interaction as realistically as possible.

Male and female subjects were included if they were healthy and had a body mass between 60 and 100 kg. This selection aimed to cover an average body mass roughly equivalent to the usual weight of aircraft passengers of 88 kg in the EU airspace [34]. Twelve subjects (4 female/8 male, 38 ± 7 years) with an average height of 1.78 ± 0.10 m and a body mass of 82.4 ± 11.7 kg were selected to meet the inclusion criteria. All participants voluntarily participated in the study and provided written informed consent. The study was conducted in accordance with the Declaration of Helsinki.

The studied structure was represented by two elastic floor panels with a size of 900 × 501 × 10 mm. They differed only in their weight-specific bending stiffness due to their composite structure. Nine panels were placed in series on an 8.10 m long structural frame made of aluminium profiles. The floor panel under investigation was placed on the middle construction frame, which was connected to a force plate (Kistler, type 9287BA, Winterthur, Switzerland). In order to take into account the overall influence of the structural bearing, the panels were either cantilevered or load-bearing. Since the gait speed of the subjects has a considerable influence on the GRF measurement results [35], two light barriers (ALGE-TIMING GmbH, ALGE photocell RLS1c, Lustenau, Austria) with a distance of 3.18 m were used to record the gait speed. All subjects walked at a first speed in the range of 1.3 to 1.5 m s^−1^ and at a second speed in the range of 1.5 to 1.7 m s^−1^ through the mock-up cabin corridor. The test persons wore either casual shoes with soft outer soles or business shoes with hard outer soles. Under the same boundary conditions, three measurements were performed by each test person; 576 measurements were recorded, i.e. 48 per subject. The sampling rate of the force plate was 1000 Hz. The GRF measured by the force plate were exported to Matlab for further data processing.

### 2.3. Processing experimental data

Measured experimental GRF data were low-pass filtered using a fourth-order Butterworth filter with a cut-off frequency of 67 Hz. The corresponding GRF results from the SLIP model simulation were converted to the experimental sampling rate to allow a direct comparison in statistical analysis. Significant GRF values $F_{v,\max}$, $F_{v,\min}$, $F_{ap,\max}$ and contact time $t_{c}$ were extracted from the trial GRF, where $F_{v,\max}$ and $F_{ap,\max}$ represent the maximum absolute value of the vertical and anteroposterior GRF, respectively (cf. figure 2*b*):

2.9 $$\begin{array}{c} F_{v,\max}=\max\left\{F_{v,1},\;F_{v,2}\right\},\\ F_{ap,\max}=\max\left\{\left|F_{ap,1}\right|,\;\left|F_{ap,2}\right|\right\}. \end{array}$$

The model leg length $l_{0}$ was calculated considering the leg-to-body ratio

2.10 $$A_{lh}=\frac{h_{0}}{l_{0}},$$

where $h_{0}$ describes the body height. Concrete values of $A_{lh}$ were measured by Kim & Bertram [36] who assumed the anatomical leg length. Using a method by Lipfert [37], these values could be converted to calculate the resulting model leg length. The results gave an overall $A_{lh}$ of 1.78 for women and 1.70 for men.

The parameters $v_{G}$ and $F_{v,\min}$ served as inputs for the simulation and calibration of the SLIP model. Values of the user mass $m_{0}$ and height $h_{0}$ were not necessary as direct input due to the normalized SLIP model formulation. The floor panel stiffness and the panel support type were not investigated separately to keep the number of input parameters as small as possible. Different shoe types were only important for the load case identification of aircraft user classes. The apparent boundary conditions nevertheless helped to reproduce the conditions in the aircraft interior as a whole.

Since the experimental data were used in two steps for model calibration and prediction, the entire amount of data was split into two-thirds for training and one-third for testing, to avoid overfitting [38]. The training dataset contained 365 trials, while the test dataset contained 185 trials. Both added up to 550 trials that were analysed in this study. Twenty-six datasets were excluded due to an erroneous or incomplete GRF recording.

### 2.4. Analysing data for model calibration

Two parameter constraints were introduced to compare results from the SLIP model simulation with experimental data. Constraint V assumed equal values of $v_{G}$ in simulation and experiment, i.e. similar locomotion kinematics. In constraint F, equal $F_{v,\min}$ was assumed representing similar kinetics. These two particular constraints were chosen because they could be easily taken from experimental data and directly specified for the SLIP model simulation. To account for limited system dynamics in the SLIP model, it was assumed that the model contact time $t_{c,\mathrm{sim}}$ was not the same as in the experiment ($t_{c,\exp}$) [20]. Therefore, the contact time factor $A_{tc}$ was introduced to quantify the deviations,

2.11 $$A_{tc}=\frac{t_{c,\exp}}{t_{c,\mathrm{sim}}}.$$

This factor facilitates the compensation of possible limitations of the SLIP model compared with experimental data. On the other side, the factor $A_{tc}$ is a simple but effective measure to quantify these limitations and to serve as a basis for further investigations on model improvements.

The initial conditions of the SLIP model simulation were defined at the VLC. $F_{v,\min}^{0}$ was assumed to equal $F_{v,\min}$ and the vertical speed ${\dot{y}}_{0}^{0}$ to be zero, so that symmetric GRF curves were generated [28].

A search algorithm was applied to find the best-fitting model parameters with a fixed $v_{G}$ (constraint V) or $F_{v,\min}$ (constraint F), which were taken as inputs from experiment. For this purpose, the parameter range was evenly divided (cf. table 3) and searched by parameter sweep. The parameter space was further reduced by linking $\varphi_{TD}$ to ${\tilde{v}}_{G}$ as suggested by Kim & Park [13],

2.12 $$\varphi_{TD}({\tilde{v}}_{G})=-0.262\;{\tilde{v}}_{G}-0.180.$$

**Table 3.** Definition and domain of parameters for the bipedal model simulation.

parameter symbol	description	determination	domain
${\tilde{m}}_{0}$	point mass (CoM)	normalization	${\tilde{m}}_{0}=1$
${\tilde{l}}_{0}$	leg length	normalization	${\tilde{l}}_{0}=1$
${\tilde{r}}_{0}$	roller foot radius	normalization	${\tilde{r}}_{0}=0.3$
${\tilde{k}}_{0}$	leg spring stiffness	via $\varphi_{TD}$ and $p_{\varphi k}$	${\tilde{k}}_{0}\in (0,317]$
${\tilde{v}}_{G}^{0}$	initial gait speed	by means of grid search algorithm	${\tilde{v}}_{G}^{0}\in [0.15,0.8]$
${\tilde{F}}_{v,\min}^{0}$	initial vertical GRF (trough value)	by means of grid search algorithm	${\tilde{F}}_{v,\min}^{0}\in (0,1.2]$
$\varphi_{TD}$	touchdown angle at FC condition	via ${\tilde{v}}_{G}$	$\varphi_{TD}\in {\left[12.6,22.3\right]}^{\circ }$
$p_{\varphi k}$	step parameter	by means of grid search algorithm	$p_{\varphi k}\in (0,10.5]$
${\tilde{t}}_{c}$	contact time with ground (stance phase)	via leg activation	–

**Figure 4.**
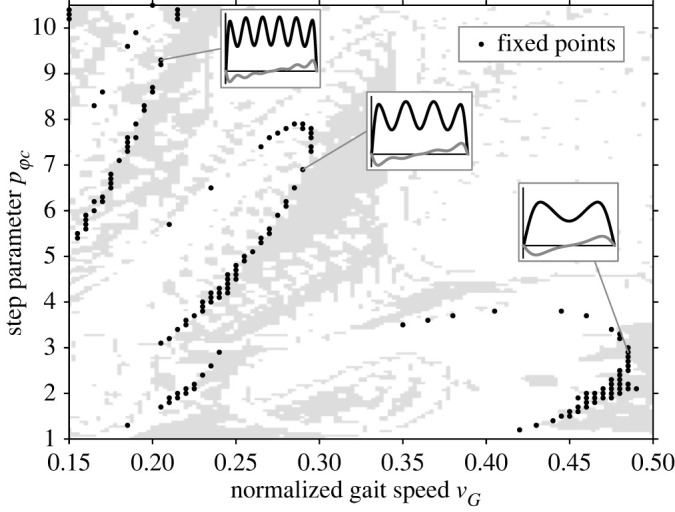
Sample of the Poincaré return map for constraint F (${\tilde{F}}_{v,\min}^{0}=0.6$) as a search space for model calibration. The domains of ground reaction force patterns are highlighted; parameter areas with a locally stable locomotion are shaded grey. Each domain of ground reaction force patterns is marked by a sample curve including values in vertical (black) and anteroposterior (grey) direction. The common oscillation domain incorporates only two main peaks in the vertical ground reaction force (right pattern).

Fixed points $z_{p}^{*}$ were determined via equation (2.5) and a limit value of $\Delta z_{p,\lim}=10^{-3}$. Among these, the largest SLIP model step length $l_{s,\mathrm{sim}}$ led to the final choice of the model configuration. Figure 4 shows an exemplary search space for constraint F at ${\tilde{F}}_{v,\min}^{0}=0.6$. Fixed points $z_{p}^{*}$ are marked as dots; areas of local stability are shaded grey. The stability area has obviously many small parts due to the underlying discrete parameter grid. The fixed points are grouped into different domains characterized by similar GRF shapes (cf. figure 4). Largest values of $l_{s,\mathrm{sim}}$ were required to generate GRF preferably in the domain of common double-hump patterns. It was not possible for a large part of the subject trials to set both $v_{G}$ and $F_{v,\min}$ from the experiment as initial conditions for the SLIP model. Thus, only letting one of the parameters be equal for each constraint seemed reasonable.

After calculating the normalized results from the SLIP model simulation, the vertical and anteroposterior GRF as well as the contact time were re-dimensioned using the body mass $m_{0}$ and the body height $h_{0}$ together with equation (2.10). These values were then compared with experimental measurements from the training dataset, yielding absolute and relative errors for $t_{c}$, $F_{v,\max}$, $F_{ap,\max}$, $F_{v,\min}$ and $v_{G}$.

All SLIP model parameters are listed in table 3. It includes methods for the value determination as well as ranges for parameter sweep.

Two additional statistical models were used to assist the GRF prediction by means of the SLIP+ model. On the one hand, the contact time factor $A_{tc}$ was analysed. On the other hand, constraint F requires values of $F_{v,\min}$ as an input. Both parameters were modelled with the user-related input parameters $v_{G}$, $h_{0}$ and $m_{0}$ as predictor variables. Direct regression models between the user-related input parameters ($v_{G}$, $h_{0}$ and $m_{0}$) and GRF outputs ($t_{c}$, $F_{v,\max}$ and $F_{ap,\max}$) were also calibrated. These models were used as a reference to assess the prediction capabilities of the SLIP+ model-based GRF prediction. For each statistical relationship, general linear models as well as the mean value over all results, i.e. constant values independent from input variables, were considered as a first approach. The step length $l_{s}$ was incorporated as an additional predictor variable via $l_{s}∼\sqrt{v_{G}}$ [39] to improve parameter predictions which strongly depend on $l_{s}$. Because the GRF parameters $F_{v,\max}$, $F_{ap,\max}$ and $F_{v,\min}$ are already normalized in terms of the body mass $m_{0}$, no mass-related coefficient was assumed here.

All calibration models and errors were evaluated by a statistical analysis. The regression coefficients and their confidence intervals of 95% were calculated. The coefficient of determination $R^{2}$ and the normalized root mean square error (NRMSE) indicated the accuracy of the proposed relationships. When normalizing the errors, the interquartile range $Q_{3}-Q_{1}$ was considered to avoid a strong dependence on outliers.

### 2.5. Analysing data for ground reaction force prediction

The GRF were predicted from SLIP+ model simulations in terms of the parameter constraints V and F as well as from direct regression models as a reference. Only the easily estimable user-related parameters gait speed $v_{G}$, body height $h_{0}$ and body mass $m_{0}$ served as inputs to the SLIP+ model prediction. The SLIP+ model predicted full vertical and anteroposterior GRF curves and the gait speed $v_{G}$. The factor $A_{tc}$ scaled the generated model GRF curve in time. The statistical representation of the GRF trough value $F_{v,\min}$ served as an input for the SLIP+ model simulation under constraint F. The most appropriate statistical models were chosen regarding high values of $R^{2}$, low values of the NRMSE, and low coefficient uncertainties. Experimental test data were used for validating the GRF prediction. The errors were determined in terms of mean, range and standard deviation (s.d.) for both constraints V and F as well as the direct regression models as a reference.

## 3. Results

### 3.1. Experiments

**Figure 5.**
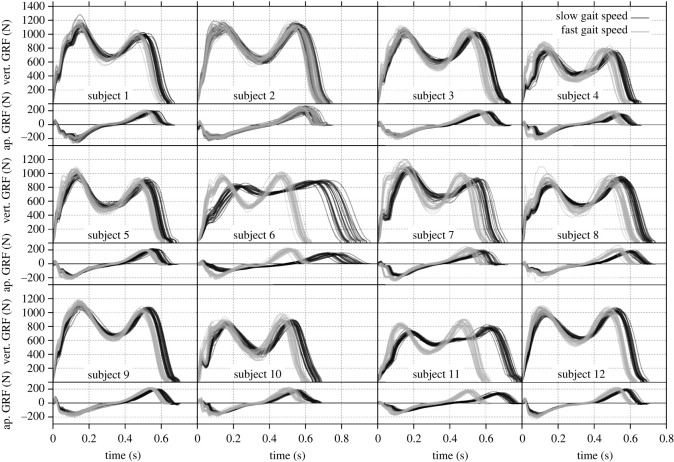
Ground reaction forces from experiments representing the entire dataset. Vertical (vert.) and anteroposterior (ap.) forces are displayed for each subject. Slow (1.3–1.5 m s^−1^) and fast (1.5–1.7 m s^−1^) gait speeds are indicated by dark and light lines, respectively.

Figure 5 shows the vertical and anteroposterior GRF for all subject trials. Different gait speed ranges, as defined in the experimental protocol, have been highlighted. Slower gait speeds come along with longer contact times $t_{c}$, lower maximum GRF $F_{v,\max}$ and $F_{ap,\max}$, and higher GRF trough values $F_{v,\min}$ for nearly all subjects. The GRF follow mostly similar curves. The largest variations occur at the beginning of the stance phase where high-frequency components emerge. Furthermore, table 4 lists the according subject parameters. Mean values and standard deviations are included for both speed ranges.

**Table 4.** Subject parameters and gait speed measured from experiment. Gait speed is further divided into slow and fast walking. Each gait speed value is displayed with one standard deviation on each side.

subject	$m_{0}$ (kg)	$h_{0}$ (m)	$v_{G}$ (m s^−1^)	$v_{G,\mathrm{slow}}$ (m s^−1^)	$v_{G,\mathrm{fast}}$ (m s^−1^)
1	97.7	1.76	1.46 ± 0.11	1.39 ± 0.05	1.59 ± 0.04
2	95.6	1.89	1.50 ± 0.07	1.48 ± 0.07	1.51 ± 0.07
3	85.1	1.95	1.50 ± 0.09	1.41 ± 0.03	1.58 ± 0.04
4	61.6	1.58	1.48 ± 0.15	1.33 ± 0.03	1.62 ± 0.04
5	77.3	1.67	1.58 ± 0.07	1.52 ± 0.05	1.64 ± 0.04
6	82.5	1.83	1.28 ± 0.34	0.94 ± 0.05	1.61 ± 0.06
7	88.3	1.76	1.39 ± 0.20	1.22 ± 0.03	1.57 ± 0.13
8	75.3	1.74	1.43 ± 0.16	1.27 ± 0.03	1.58 ± 0.04
9	95.7	1.85	1.56 ± 0.07	1.49 ± 0.03	1.63 ± 0.02
10	72.1	1.73	1.48 ± 0.13	1.35 ± 0.03	1.59 ± 0.04
11	68.2	1.70	1.37 ± 0.25	1.13 ± 0.03	1.61 ± 0.04
12	89.3	1.84	1.54 ± 0.09	1.45 ± 0.03	1.63 ± 0.03

### 3.2. Model calibration

Experimental results exhibit a large range of gait speed $v_{G}$ and the GRF trough value $F_{v,\min}$, which was compared with the SLIP model by means of the constraints V and F. Figure 4 shows a parameter sweep map as an example for constraint F. There are different locomotion domains depending on gait speed ${\tilde{v}}_{G}$ as already mentioned by Geyer *et al*. [9]. Moderate to high values of ${\tilde{v}}_{G}$ feature a common vertical GRF curve with two peaks while low speeds of about ${\tilde{v}}_{G}<0.3$ seem to incorporate higher oscillation modes with multi-peaked GRF curves (cf. figure 4). Experimental values of ${\tilde{v}}_{G}$ cover an interval from 0.26 to 0.57, which means that most of the trials lie within the common oscillation domain with two main peaks in the vertical GRF.

The SLIP model described in §2.1 requires two additional statistical models to compensate for model limitations and to predict GRF without further experimental data. These statistical models estimate the parameters ${\tilde{F}}_{v,\min}$ and $A_{tc}$ in relation to the inputs $v_{G}$, $h_{0}$ and $m_{0}$ (cf. §2.4). The relationships for ${\tilde{F}}_{v,\min}$ and $A_{tc}$ can be found in table 5 along with the regression results. All proposed relationships show nearly equal $R^{2}$ and NRMSE. The second model, which only depends on $\sqrt{v_{G}}$ instead of $v_{G}$, was chosen because the plausible value of ${\tilde{F}}_{v,\min}(v_{G}=0)=1$ was additionally achieved. A constant value for $A_{tc}$ was chosen for constraint V due to a low $R^{2}$. The linear relationship for $A_{tc}$ regarding constraint F has a clearly higher $R^{2}$.

**Table 5.** Possible statistical regression models for parameter prediction. All coefficients were calculated based on experimental training data. The time scaling factor $A_{tc}$ incorporates both results from experiment and walking model simulation. For all other parameters only experimental data were used. General mathematical forms as well as mean coefficient values, coefficient of determination and NRMSE (confidence interval of 95%) are displayed; the regression models selected for prediction are highlighted grey. Dimensions are neglected for better readability.

predicted parameter	general form	$c_{4}$	$c_{3}$	$c_{2}$	$c_{1}$	$c_{0}$	$R^{2}$	NRMSE
$A_{tc}$(constraint V)	$c_{0}$	—	—	—	—	1.531 ± 0.007	—	0.621
	$c_{3}\;v_{G}+c_{2}\;h_{0}+c_{1}\;m_{0}+c_{0}$	—	0.0164 ± 0.0347	−0.126 ± 0.093	−0.00166 ± 0.00085	1.868 ± 0.135	0.193	0.561
$A_{tc}$(constraint F)	$c_{0}$	—	—	—	—	1.461 ± 0.013	—	0.881
	$c_{3}\;v_{G}+c_{2}\;h_{0}+c_{1}\;m_{0}+c_{0}$	—	−0.436 ± 0.055	0.113 ± 0.150	−0.000910 ± 0.001358	1.973 ± 0.215	0.414	0.677
$t_{c}$	$c_{3}\;v_{G}+c_{2}\;h_{0}+c_{1}\;m_{0}+c_{0}$	—	−0.298 ± 0.017	0.297 ± 0.046	−0.000652 ± 0.000419	0.627 ± 0.067	0.802	0.407
	$c_{3}\sqrt{v_{G}}+c_{2}\;h_{0}+c_{1}\;m_{0}+c_{0}$	—	−0.696 ± 0.038	0.292 ± 0.045	−0.000648 ± 0.000407	1.034 ± 0.076	0.813	0.395
	$c_{4}\;v_{G}+c_{3}\sqrt{v_{G}}+c_{2}\;h_{0}+c_{1}\;m_{0}+c_{0}$	−3.246 ± 0.790	1.104 ± 0.341	0.280 ± 0.043	0.000483 ± 0.000389	2.510 ± 0.462	0.832	0.375
${\tilde{F}}_{v,\max}$	$c_{2}\;v_{G}+c_{1}\;h_{0}+c_{0}$	—	—	0.212 ± 0.032	−0.337 ± 0.058	1.523 ± 0.112	0.446	0.606
	$c_{2}\sqrt{v_{G}}+c_{1}\;h_{0}+c_{0}$	—	—	0.490 ± 0.074	−0.336 ± 0.058	1.240 ± 0.134	0.446	0.605
	$c_{3}\;v_{G}+c_{2}\sqrt{v_{G}}+c_{1}\;h_{0}+c_{0}$	—	0.529 ± 1.615	−0.0171 ± 0.6985	−0.336 ± 0.058	1.217 ± 0.937	0.446	0.606
${\tilde{F}}_{ap,\max}$	$c_{2}\;v_{G}+c_{1}\;h_{0}+c_{0}$	—	—	0.111 ± 0.014	−0.171 ± 0.025	0.386 ± 0.048	0.537	0.506
	$c_{2}\sqrt{v_{G}}+c_{1}\;h_{0}+c_{0}$	—	—	0.258 ± 0.032	−0.170 ± 0.025	0.237 ± 0.058	0.537	0.506
	$c_{3}\;v_{G}+c_{2}\sqrt{v_{G}}+c_{1}\;h_{0}+c_{0}$	—	0.143 ± 0.697	0.0495 ± 0.3014	−0.170 ± 0.025	0.303 ± 0.405	0.537	0.507
${\tilde{F}}_{v,\min}$	$c_{2}\;v_{G}+c_{1}\;h_{0}+c_{0}$	—	—	−0.346 ± 0.026	0.146 ± 0.048	0.919 ± 0.093	0.658	0.520
	$c_{2}\sqrt{v_{G}}+c_{1}\;h_{0}+c_{0}$	—	—	−0.800 ± 0.061	0.146 ± 0.048	1.380 ± 0.111	0.659	0.519
	$c_{3}\sqrt{v_{G}}+c_{2}\;v_{G}+c_{1}\;h_{0}+c_{0}$	—	−0.745 ± 1.333	−0.0238 ± 0.5765	0.146 ± 0.048	1.348 ± 0.774	0.659	0.520

Table 6 displays all relative calibration errors when only the SLIP model is used without any additional regression models. The clear underestimation of the contact time $t_{c}$ of more than 30% is most noticeable, as expected from findings of Lipfert *et al*. [20]. The SLIP model locomotion tends to produce a lower maximum vertical GRF $F_{v,\max}$ in both constraints. However, constraint V achieves the mean maximum absolute anteroposterior GRF $F_{ap,\max}$ well. The error s.d. of both GRF components is considerably higher for constraint V than for constraint F. Moreover, constraint V yields a vertical GRF trough value $F_{v,\min}$ much lower than the corresponding experimental data with high variation (error s.d.) of 26%. Similarly, constraint F underestimates the gait speed $v_{G}$.

**Table 6.** Relative regression errors from model calibration (training). Numbers with hash (^#^) are solely affected by the grid subdivision of parameter space. Gait speed was not processed by direct regression as it does not need to be predicted.

%	$t_{c}$	$F_{v,\max}$	$F_{ap,\max}$	$F_{v,\min}$	$v_{G}$
*constraint V*					
mean error	−35	−7.1	2.5	−24	0.078^#^
error standard deviation	2.9	11.5	19.1	26	0.43^#^
error range minimum	−41	−28	−37	−94	−0.98^#^
error range maximum	−25	42	78	28	1.13^#^
*constraint F*					
mean error	−31	−14.3	−12.1	0.023^#^	−7.9
error standard deviation	6.5	5.8	9.6	0.85^#^	12.3
error range minimum	−45	−26	−36	−2.0^#^	−46
error range maximum	−4.5	12	28	1.65^#^	25
*direct regression*					
mean error	0.20	0.91	0.167	0.57	—
error standard deviation	4.5	9.7	4.3	8.0	—
error range minimum	−13.5	−26	−11.2	−13.6	—
error range maximum	14.1	34	16.7	40	—

Table 5 further shows all suggestions for possible direct regression models for contact time $t_{c}$, vertical GRF ${\tilde{F}}_{v,\max}$ and anteroposterior GRF ${\tilde{F}}_{ap,\max}$ as a function of the user-related input parameters. The direct regression model only depending on $\sqrt{v_{G}}$ was chosen for $t_{c}$ because the regression model with both predictors $v_{G}$ and $\sqrt{v_{G}}$ had high deviations in the coefficients $c_{3}$ and $c_{4}$. All possible regression models for ${\tilde{F}}_{v,\max}$ showed nearly the same $R^{2}$ and NRMSE. Finally, the model only depending on $v_{G}$ was selected because it resembled the plausible value of ${\tilde{F}}_{v,\max}(v_{G}=0)=1$ well. For ${\tilde{F}}_{ap,\max}$, the regression model with the least NRMSE was chosen.

All direct regression models show very good relative mean errors in table 6. Variation in terms of the error s.d. lies in the same magnitude as the constraints V and F for the parameters $t_{c}$ and $F_{v,\max}$. The error s.d. of $F_{ap,\max}$ and $F_{v,\min}$ is clearly lower than that of the SLIP model-based simulations.

### 3.3. Errors of prediction

**Figure 6.**
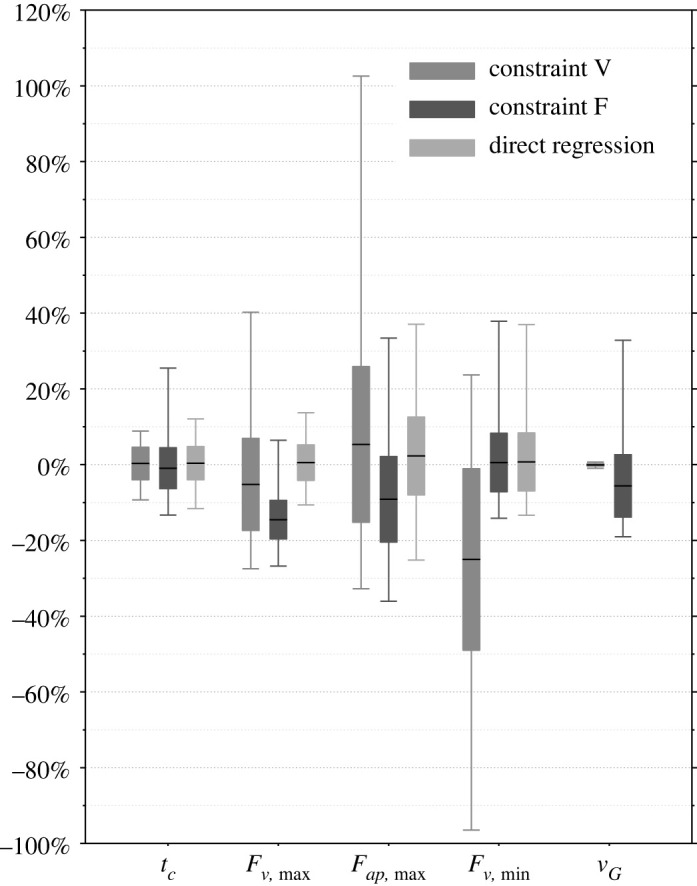
Prediction errors of the bipedal model simulation for constraints V and F as well as of direct regression. All values were calculated based on experimental test data: The error s.d. level is represented by boxes, whiskers stand for the entire error range, mean values are marked by black dashes. Gait speed was not predicted by direct regression.

Figure 6 shows the model prediction errors for both constraints and the direct regression models as a reference. Constraint V predicts the mean GRF values $F_{v,\max}$ and $F_{ap,\max}$ well with an error of −5.2% and 5.3%, respectively. However, the deviations of the force parameters $F_{v,\max}$ and $F_{ap,\max}$ with respect to the error s.d. are comparatively large, indicating a weak predictability. Constraint F allows a more reliable prediction regarding these two force parameters with an error s.d. of 5.1% and 11.3%, respectively. The direct regression models exhibit similar results in this case with 4.7% s.d. for $F_{v,\max}$ and 10.3% s.d. for $F_{ap,\max}$. For constraint F, the error of the maximum vertical force $F_{v,\max}$ lies in an almost completely negative range from −27% to 6.5%, indicating a well-treatable upper limit of the structural load. Constraint V predicts the contact time $t_{c}$ with a lower variation (error s.d.) of 4.3%, similar to but even slightly lower than 4.4% estimated by direct regression, while constraint F shows a weaker prediction with 5.4% s.d. All error-related values for $F_{v,\min}$ are very similar between constraint F and the direct regression model as constraint F uses theoretically the same statistical model. The mean error of the direct regression models lies near zero for all estimated parameters.

On the one hand, constraint V has a very good data fit for initially equal $v_{G}$, while lacking accuracy in predicting $F_{v,\min}$. On the other hand, constraint F shows a good prediction for $F_{v,\min}$ and achieves $v_{G}$ with a low mean error of −5.6%. Both constraints show similar error s.d. as the direct regression models, especially for force-related values in constraint F.

**Figure 7.**
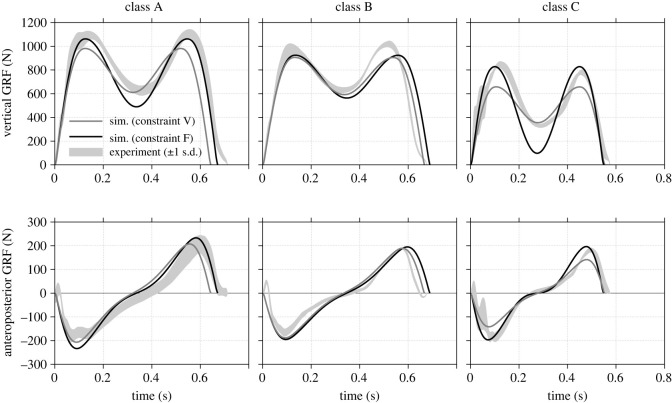
Bipedal model predictions of ground reaction force (GRF) curves in terms of different aircraft user classes. Mean GRF predictions (solid grey line: constraint V, solid black line: constraint F) are compared with experimental curves from the test dataset in the limits of one standard deviation on each side (grey shaded area). Class A: tall height, heavy weight, fast gait speed. Class B: average height, average weight, normal gait speed. Class C: small height, low weight, fast gait speed.

In order to demonstrate the capabilities of the SLIP+ model for predicting load cases for structural design, critical aircraft user classes were specified from three distinct load cases: first, a tall and heavy person walking fast over the floor structure represents the highest expectable single load in operation (A). Second, a person with average size and a normal gait speed stands for a common load case which is important for life cycle analysis of the floor structure (B). Third, a rather small woman walking fast over the floor probably wears high heels with a small heel base size. This type of shoe induces a high pressure on the floor and hence the highest expectable stresses in the structure (C). Three subjects were chosen from the trial data as listed in table 7 in order to represent each class. Figure 7 displays the results for all user classes. Both the mean GRF curve which was predicted by the SLIP+ model with the constraints V and F as well as the corresponding experimental GRF curves from the test data are shown. Classes A and B show good agreement with the experimental data. Constraint V exhibits good matches for the maximum GRF in vertical and anteroposterior direction while underestimating the trough value of the vertical GRF. On the other hand, constraint F cannot reach the GRF maxima exactly and keeps a comparatively lower force amplitude. Class C shows prediction results that diverges more: constraint V predicts a clearly lower trough value in the vertical GRF, whereas constraint F has too low values for the maxima. It seems that constraint V reaches a better prediction for the critical maximum GRF values than constraint F for all classes. Furthermore, the predictions do not reproduce high-frequency components shown in experimental GRF data especially at the beginning of the stance phase (see also figure 5).

**Table 7.** Aircraft user classes with the representing test subject. Each class stands for a type of criticality regarding the design of aircraft interior floor panels: subject 2 as a tall and heavy person walking fast; subject 12 with normal body size, weight and gait speed; subject 4 as a rather small woman probably wearing high heels. Gait speed is displayed with one standard deviation on each side and is taken from experimental test data.

user class	subject	$v_{G}$ (m s^−1^)	${\tilde{v}}_{G}$	criticality
A	2	1.58 ± 0.03	0.471 ± 0.009	single load
B	12	1.45 ± 0.03	0.445 ± 0.010	fatigue load
C	4	1.63 ± 0.05	0.552 ± 0.017	heel pressure

## 4. Discussion

In this study, a SLIP model with roller feet helped to predict GRF for load cases of user–structure interactions in the aircraft interior. Human gait experiments were carried out in an aircraft mock-up to resemble common environmental conditions. Two parameter constraints V and F were proposed to compare the SLIP model simulations with experimental data in a representative way. Additional statistical models were included to compensate for SLIP model limitations. A parameter sweep found appropriate model parameters and resulted in simulated GRF in vertical and anteroposterior direction. These GRF were analysed in terms of user classes which stand for critical load cases in the design of floor structures. Direct statistical regression models of significant GRF parameter values served as a reference.

Previous studies suggest a model–experiment comparison via fitting various walking model parameters to experimental values. These can be kinematic parameters such as gait speed $v_{G}$ [20,21], CoM trajectory $x_{0}(t)$ [19], or touchdown angle $\varphi_{TD}$ [20]. Furthermore, leg parameters, e.g. for anatomical spring-like and damping function, are compared with experiments [13,20,24]. This paper presents two constraints V and F that serve to specify initial walking conditions for the SLIP model clearly and easily in terms of gait speed $v_{G}$ and the GRF-related parameter $F_{v,\min}$, respectively. With it, SLIP model limitations may be better analysed and understood than with overall parameter optimization. Besides maximal GRF values, which are critical for structural design, contact time $t_{c}$ could be consequently predicted with low errors by means of the scaling factor $A_{tc}$.

### 4.1. Limitations of the SLIP model

As can be seen from table 6, the results from SLIP model simulations show acceptable mean errors of GRF for constraint V, while constraint F underestimates all maximal force values $F_{v,\max}$ and $F_{ap,\max}$. Nevertheless, contact time $t_{c}$ exhibits good predictability in terms of the error s.d. for both constraints, especially constraint V with a value even less than that of the direct regression model. Error s.d. values of GRF are better for constraint F lying in the same order of magnitude as the direct regression models. These results indicate that the GRF prediction by means of the SLIP model can basically be carried out with similar reliability (error s.d.) as the statistical approach without the SLIP model.

The most remarkable deviations are that for the contact time $t_{c}$ reaching values of up to −35%. It can be supposed that similar limitations are apparent in the SLIP templates of Geyer *et al*. [9] and Whittington & Thelen [12] used throughout this paper. As the factor $A_{tc}$ expresses the errors of $t_{c}$, it can be used to quantify these system limitations in comparison with other SLIP models. Average values of the SLIP model used in this study are 1.53 and 1.46 for constraints V and F, respectively. By contrast, the simplest SLIP template without roller feet reaches values for $A_{tc}$ of 1.55 and 1.21 according to own analyses. The value of Lipfert *et al*. [20] can be estimated as around 1.5 to 1.6. Hence, both SLIP models seem to have similar limitations for constraint V. On the other hand, constraint F exhibits a lower $A_{tc}$ for the simplest SLIP template than for the template used in this study. Because constraint F requires similar kinetics in form of $F_{v,\min}$, lower $A_{tc}$ can be ascribed to the different formulation of the leg spring. In the SLIP model of Whittington & Thelen [12] the leg spring is relatively shorter and is thus limited to generate only a smaller spring compression *s*. This is assumed to induce low GRF amplitudes for constraint F observed in figure 7.

Another important limit for the SLIP model is that the model dynamics exhibit higher oscillation modes for low $v_{G}$ [9]. Looking at figure 4, the limit is at about ${\tilde{v}}_{G}=0.3$. This speed limit is called the inflection point by Smith & Lemaire [40] and is associated with different gait kinematics. The authors explain the differences with a more active gait at low $v_{G}$, which causes a higher variability in the gait parameters. Although higher oscillation modes have also been observed in real human walking [31,41] and even during bird terrestrial locomotion [42], the results of an entirely passive walking model consequently have to be less accurate. From this study, few experimental values of ${\tilde{v}}_{G}$ are below the inflection point, as described in §3.1. In particular, the deviations of these results obtained for constraint F are suspected to be high, as the definition of ${\tilde{F}}_{v,\min}$ assumes only one trough in the common vertical GRF curve. Possible higher oscillation modes in the experimental GRF are thus neglected.

Second, the model gait speed $v_{G}$ has an upper limit [20]. That means the model cannot reproduce all values of gait speed from the subject trials. Own analyses estimate the limits to be about ${\tilde{v}}_{G}=0.5$ for ${\tilde{r}}_{0}=0$ and ${\tilde{v}}_{G}=0.6$ for ${\tilde{r}}_{0}=0.3$. The CoP excursion induced by roller feet seems to allow a higher possible gait speed in model locomotion. Constraint V is based on the same $v_{G}$ for model and trial locomotion. Due to the upper limit for gait speed, not every trial can be considered in this constraint and the accuracy of the model simulation parameters is affected. Large deviations with very small values for the GRF parameter $F_{v,\min}$ represent inappropriate system dynamics at this speed limit. The reason for this problem may lie in the properties of the passive legs: Buczek *et al*. [19] identify an active push-forward just before FO in real human gait to allow wider steps and higher $v_{G}$.

Further reasons for apparent limitations in the model dynamics could be as follows:

(1) The touchdown angle $\varphi_{TD}$ is too small and the relation $\varphi_{TD}({\tilde{v}}_{G})$ in equation (2.12) does not fit to the actual model–experiment comparison.
(2) Circular roller feet, as part of a simple gait model template, do not describe the CoP excursion precisely enough. Therefore, more elaborate foot models are necessary.
(3) The roller feet shorten the leg springs inappropriately. Hence, the application point of the spring should lie in the CoP. Longer leg springs could also be achieved by the concept of the virtual pivot point (VPP) identified by Maus *et al*. [10]. The VPP lies above CoM and is pointed to by the GRF throughout the stance phase. Blickhan *et al*. [43] apply this concept to a model in which the spring legs point to the virtual pivot point. Similar concepts of virtually extended legs are mentioned by Alexander [7] to accommodate to GRF and by Gard & Childress [14] in terms of a subsurface virtual walking surface.
(4) During walking, real human legs exhibit a non-constant virtual leg stiffness $k_{0}$. As shown by Lipfert *et al*. [20], real values for $k_{0}$ are highly variable under compression. The parameter $k_{0}$ only represents an average value, which has lower errors for faster $v_{G}$. Riese & Seyfarth [44] further investigate possible variations in the parameters $k_{0}$ and $l_{0}$ over contact time $t_{c}$ for human hopping. A positive ${\dot{l}}_{0}$ and negative ${\dot{k}}_{0}$ reproduces a realistic leg function in the loading response phase (after FC) as well as in the active push-forward phase (before FO). Although the authors only considered leg stiffness for hopping, the results can easily be transferred to human gait. By varying $k_{0}$ and $l_{0}$, energy loss is avoided so that gait remains a passive locomotion [44]. In addition, Rummel & Seyfarth [18] propose a slightly different leg model that uses knee joints with clock springs as a more complex model for the linear leg spring template. The authors state that the overall leg stiffness $k_{0}$ varies with respect to knee flexion and moment, making the force–length relationship of the usual leg spring template nonlinear.

Despite the model limitations listed above, the contact time $t_{c}$ for both constraints and all GRF values for constraint F remain predictable due to the low error s.d. as illustrated by table 6. This serves as a basis for using the contact time factor $A_{tc}$ as a reliable measure of SLIP model limitations as well as for compensatory statistical regression models. It is remarkable that error s.d. values of the contact time $t_{c}$ with constraint V stay lower than that of the direct regression model. At this point, results from the SLIP model seem to have the potential to be more predictable than from simple statistical models.

### 4.2. Predicting load cases without experiments

The SLIP+ model predicted GRF curves without the need for further experimental data for different aircraft user classes (cf. figure 7). Together with additional regression models, the contact time $t_{c}$ could be predicted very well for all simulations. However, prediction qualities regarding the GRF values $F_{v,\max}$, $F_{ap,\max}$ and $F_{v,\min}$ were different for each constraint V and F. Constraint V generated well-fitting maximum GRF values, while the amplitude stayed too high to properly predict the vertical GRF trough value $F_{v,\min}$. Especially user class C had a high normalized gait speed ${\tilde{v}}_{G}$ (cf. table 7), which lies close to the SLIP model limit of about ${\tilde{v}}_{G}=0.6$ mentioned in the previous section. The highest possible gait speed ${\tilde{v}}_{G}$ for constraint V brings $F_{v,\min}$ to zero, so that the prediction errors of $F_{v,\min}$ are particularly high at fast $v_{G}$. At the same time, $F_{v,\min}$ was estimated better by constraint F. However, the GRF amplitude stayed lower at slower gait speeds $v_{G}$, so that maximum GRF values could not be predicted in the same quality as constraint V.

Constraint V was superior to constraint F in predicting the GRF maxima $F_{v,\max}$ and $F_{ap,\min}$ due to a higher GRF amplitude as stated above. Nevertheless, two shortcomings regarding the prediction of these parameters could be identified. First, GRF peak values are slightly shifted in the phase of load response, as can be seen from figure 7. This proves a slight misalignment of the model GRF vector. A reason for that could be that real GRF are measured at the CoP, whereas the GRF vector from the leg spring of the SLIP model simulation does not point exactly there. Second, Rummel *et al*. [28] find that a positive initial vertical speed ${\dot{y}}_{0}^{0}$ leads to higher first peaks in the vertical GRF. This force pattern could probably occur in real human gait during active push-forward as identified for user class C. However, the current results of the SLIP+ model simulation cannot represent asymmetric vertical GRF as ${\dot{y}}_{0}^{0}$ is set to zero at the VLC.

Both constraints V and F can finally be assessed in terms of how appropriate they are as a GRF predictor in an end-to-end design process. While the SLIP+ model could predict the GRF for user classes A and B well, class C offered larger prediction errors. That suggests that high gait speeds cannot be represented appropriately by either of the constraints due to the limited gait speed in the SLIP model. Constraint V could generally better predict maximum GRF values $F_{v,\max}$ and $F_{ap,\max}$, which are seen as critical parameters for structural design. The overall prediction quality is represented by a low error s.d. value for GRF. Constraint F exhibits considerably lower values regarding GRF-related parameters than constraint V (cf. figure 7) providing a more reliable basis for the estimation of structural load cases. Studies by Ryu & Park [21] show error s.d. values in the same order of magnitude for GRF prediction with only experimental GRF data available. These results indicate a common level of error in the present prediction method. Therefore, the higher prediction reliability speaks in favour of constraint F.

Moreover, it has to be evaluated if a SLIP model is necessary for accurate GRF prediction or if direct parameter regression models should be preferred. Looking at the results of the direct regression models, the error levels are similar to the best results of the SLIP+ model simulation. Especially $F_{v,\min}$ has the same values for constraint F and the direct regression because constraint F depends on the regression model for $F_{v,\min}$ as an input parameter. Several parameters could be even better predicted by the SLIP+ model than by the direct regression model, e.g. contact time $t_{c}$ in constraint V with lower error s.d. and smaller error range. It is believed that the SLIP+ model has the potential to outperform GRF predictions by simple regression models: full GRF curves are generated together with fundamental kinematics in a dynamically consistent locomotion, while direct regression models only predict single parameter values. The results from SLIP+ model predictions give a better general view of the human-induced loads and can be applied more easily in subsequent analysis (e.g. finite-element simulations) within the structural design process. Together with errors similar to the reference prediction, SLIP+ model-based predictions are seen to be favourable in estimating load cases for structural design.

This encourages the search for further SLIP+ model improvements in order to estimate structural load cases. At first, the suggested model improvements listed in the previous section are crucial to enhance model prediction. These include adjusted model parameter values of $\varphi_{TD}$, $k_{0}$ and $l_{0}$, as well as an appropriately revised perception of circular roller feet. The SLIP model could provide more accurate results with an appropriate leg spring definition that also implies longer legs. Furthermore, longer steps could be achieved by larger possible $\varphi_{TD}$. The results of Riese & Seyfarth [44] as well as Rummel & Seyfarth [18] indicate that non-constant leg stiffness $k_{0}$ and resting leg length $l_{0}$ should vary to keep the leg behaviour realistic. A look at the contact time factor $A_{tc}$ reveals values greater than one (cf. table 5). Consequently, the model dimensions derived from the experiment must be overall increased to reproduce appropriate dynamic system behaviour. Hence, constraints V and F should finally agree, as the GRF can be realistically predicted by the gait speed $v_{G}$. Differing results from both prediction constraints show where the model concept is still inadequate.

## 5. Conclusion

In this paper, the passive SLIP model with circular roller feet according to Whittington & Thelen [12] was thoroughly investigated and compared with experimental data in order to estimate load cases for the design of aircraft interior structures. The SLIP model was reconfigured for a more general and easier formulation of the equations of motion. Two parameter constraints were introduced, newly as an explicit concept, to compare the model simulation with the experimental data in a representative, explainable way: constraint V considered equal gait speed $v_{G}$ of simulation and experiment, while constraint F ensured the same vertical GRF trough value $F_{v,\min}$. Additional statistical regression models allowed the compensation for SLIP model limitations.

In general, the critical GRF values $F_{v,\max}$ and $F_{ap,\max}$ could be predicted reliably. Single larger errors in GRF prediction were only observed for fast walking aircraft users due to gait speeds close to the system limits of the SLIP model. Very good accuracy was observed in predicting the contact time $t_{c}$. Altogether, errors were similar to that of the GRF prediction by direct regression models without SLIP model simulation.

However, direct regression models were not able to achieve considerably lower error s.d., i.e. prediction reliability was not better. It must be pointed out that the SLIP+ model predictions generate full GRF curves as well as fundamental gait kinematics while direct regression models only predict single parameters. Moreover, the SLIP+ model ensures parameter outputs that are dynamically consistent because they all come from a common gait movement. That is why the SLIP+ model is seen as more appropriate to be used within an end-to-end design process where model outputs are supposed to be used as inputs for advanced dynamic structural simulations.

The SLIP model in use exhibited initially similar limitations to the simplest SLIP template of Geyer *et al*. [9] with values for the contact time factor $A_{tc}$ of about 1.5. Together with calibrated statistical regression models, these limitations could be compensated sufficiently by the SLIP+ model. There were no further experimental data needed to predict GRF as load cases for the design of aircraft structures by a minimal set of aircraft user-related input parameters (gait speed, body height, body mass). It is believed that structural design processes will benefit from this kind of simple bipedal models like the SLIP+ model as they are easily developable, flexible and computationally efficient. Further investigations regarding the SLIP+ model proposed in this paper promise to expand the possibilities of structural design in aircraft by means of simple human models.

## Figures and Tables

**Figure 8.**
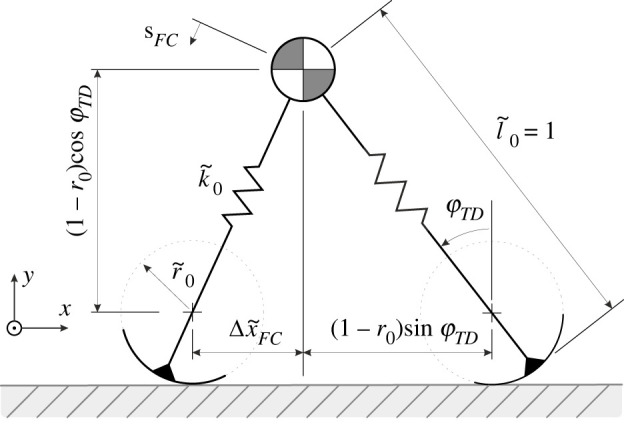
Model view at the instant of foot contact. The geometric parameter assumptions ${\tilde{s}}_{FC}$ and $\Delta {\tilde{x}}_{FC}$ are highlighted.

## Data Availability

All program code was implemented in Matlab (The MathWorks, Inc. Natick, MA, USA); figures were made with Gnuplot (https://sourceforge.net/projects/gnuplot/). Scripts and data for analysis and figure generation are provided in the electronic supplementary material [45].

## Appendix A

### A.1. Calculating locally stable system motion

In order to prove that simulation parameters discovered by parameter sweep in §2.4 are valid, local stability of the fixed points $z_{p}^{*}$ must be determined. A stable system motion is achieved if the modulus of all eigenvalues $\lambda_{p}$ of the Jacobian matrix $J_{z_{p}}$ is lower than 1 [28].

The matrix $J_{z_{p}}$ describes changes of the recurrent system state $z_{p}^{R}$ depending on the initial state $z_{p}^{0}$. State variables as defined in §2.1 are transformed into parameter values to fit to the gridded parameter domain

A 1 $$z_{p}=\left(\begin{array}{c} y_{0}\\ {\dot{x}}_{0} \end{array}\right)\to \zeta_{p}=\left(\begin{array}{c} {\tilde{v}}_{G}(y_{0},\;{\dot{x}}_{0})\\ {\tilde{F}}_{v,\min}(y_{0},\;{\dot{x}}_{0}) \end{array}\right).$$

The remaining parameter $p_{\varphi k}$ does not influence the state $z_{p}$ so that only ${\tilde{v}}_{G}$ and ${\tilde{F}}_{v,\min}$ are considered for estimating a stable locomotion. Gradients in $\zeta_{p}$ take a numerical form based on the domain grid where specific parameter values are indexed as ${\tilde{v}}_{G}(i)$ and ${\tilde{F}}_{v,\min}(j)$ with i,j=1,2,… Finally, calculating $J_{\zeta_{p}}$ yields

A 2 $$J_{\zeta_{p}}(i,j)=\left(\begin{array}{cc} \frac{{\tilde{v}}_{G}^{R}(i+1)-{\tilde{v}}_{G}^{R}(i-1)}{{\tilde{v}}_{G}^{0}(i+1)-{\tilde{v}}_{G}^{0}(i-1)} & \frac{{\tilde{v}}_{G}^{R}(i+1)-{\tilde{v}}_{G}^{R}(i-1)}{{\tilde{F}}_{v,\min}^{0}(j+1)-{\tilde{F}}_{v,\min}^{0}(j-1)}\\ \frac{{\tilde{F}}_{v,\min}^{R}(j+1)-{\tilde{F}}_{v,\min}^{R}(j-1)}{{\tilde{v}}_{G}^{0}(i+1)-{\tilde{v}}_{G}^{0}(i-1)} & \frac{{\tilde{F}}_{v,\min}^{0}(j+1)-{\tilde{F}}_{v,\min}^{0}(j-1)}{{\tilde{F}}_{v,\min}^{0}(j+1)-{\tilde{F}}_{v,\min}^{0}(j-1)} \end{array}\right),$$

with initial states ${\left(\cdot \right)}^{0}$ and return states ${\left(\cdot \right)}^{R}$ and local stability is achieved with

A 3 $$\max\lambda_{p}(J_{\zeta_{p}})<1,$$

as defined above.

### A.2. Introducing step factor to scale model parameters

The step factor $A_{tc}$ is introduced to scale down the domain of the leg stiffness parameter $k_{0}$ to an appropriate range. Seyfarth *et al*. [33] suggested a relationship for stable model locomotion that is asymptotic for small $\varphi_{TD}$. Hence, rescaling is particularly convenient for this region.

Figure 8 depicts the first FC after VLC. The following relationship

A 4 $${\left(1-{\tilde{r}}_{0}-{\tilde{s}}_{FC}\right)}^{2}=\Delta {\tilde{x}}_{FC}^{2}+{\left(1-{\tilde{r}}_{0}\right)}^{2}\cos^{2}\varphi_{TD},$$

is obvious with the leg compression ${\tilde{s}}_{FC}$ of the trailing leg. The unknown variable $\Delta {\tilde{x}}_{FC}$ is expressed in terms of

A 5 $$\Delta {\tilde{x}}_{FC}=\eta \;(1-{\tilde{r}}_{0})\sin\varphi_{TD},$$

where η∈[0,1) and η=1 for ${\tilde{s}}_{FC}=0$. Furthermore, ${\tilde{s}}_{FC}^{2}$ is small so that equation (A 4) collapses to

A 6 $$(1-{\tilde{r}}_{0})\sin^{2}\varphi_{TD}=\frac{2{\tilde{s}}_{FC}}{1-\eta^{2}}.$$

The leg compression ${\tilde{s}}_{FC}$ can be written as

A 7 $${\tilde{s}}_{FC}=\frac{{\tilde{F}}_{FC}}{{\tilde{k}}_{0}}$$

with ${\tilde{F}}_{FC}$ describing the normalized spring force of the trailing leg at the instance of FC. It is now assumed that η and ${\tilde{F}}_{FC}$ keep sufficiently constant over a certain range of $\varphi_{TD}$ and ${\tilde{k}}_{0}$. The fact that $\varphi_{TD}$ incorporates only a narrow parameter range (cf. table 3) and ${\tilde{F}}_{FC}$ keeps close to ${\tilde{F}}_{v,\min}^{0}$ embraces the stated definition. The step parameter $p_{\varphi k}$ consequently transforms into

A 8 $$p_{\varphi k}=\frac{2{\tilde{F}}_{FC}}{1-\eta^{2}}=k_{0}{\left(1-r_{0}\right)}^{2}\sin^{2}\varphi_{TD}\approx \mathrm{const},$$

serving as one of three main parameters in the parameter sweep (cf. §2.4).
